# Role of the rapid delayed rectifier K^+^ current in human induced pluripotent stem cells derived cardiomyocytes

**DOI:** 10.46439/stemcell.1.003

**Published:** 2020

**Authors:** Makarand Deo, Akwasi Akwaboah, Bright Tsevi, Jacqueline A Treat, Jonathan M Cordeiro

**Affiliations:** 1 Department of Engineering, Norfolk State University, Norfolk, Virginia, USA; 2 Department of Experimental Cardiology, Masonic Medical Research Institute, Utica, New York, USA

**Keywords:** Electrophysiology, Action potential, Repolarization reserve, Delayed rectifier, Mathematical modeling

The action potential (AP) in cardiac tissue is important for initiating and coordinating contractions in the heart. In addition, the long refractory period minimizes the potential for developing extrasystoles and arrhythmias [1]. The AP is generated by coordinate changes in different ionic currents. In human (or canine) adult ventricular cells, the depolarization phase of the AP is mainly through the influx of Na^+^ and Ca^2+^ through specific voltage gated channels [2]. Repolarization of the AP is regulated by activation of a number of different K^+^ currents which play important roles in regulating the AP. These K^+^ currents include: (i) a Ca^2+^-independent transient outward K^+^ current (I_to_), (ii) an inwardly rectifying K^+^ current (I_K1_), and (iii) the rapidly and slowly activating delayed rectifier K^+^ channel currents (I_Kr_ and I_Ks_, respectively). Previous studies have demonstrated that there is an excess of several K^+^ currents necessary for cardiac repolarization such that a net outward current remains available for repolarization if one or more currents are reduced (repolarization reserve) [3–5]. Therefore, cardiac tissue with a lower repolarization reserve is associated with a prolonged ventricular action potential and an increased incidence of developing arrhythmias [6]. Mutations in KCNH2 (the gene which encodes I_Kr_) cause a decrease in the magnitude of I_Kr_ and are associated with Long QT syndrome [7,8]. Patients afflicted with Long QT have episodes of fainting, irregular heartbeats and an increased incidence of developing ventricular arrhythmias. Interestingly, many non-cardiac medications have also been shown to block I_Kr_ [9,10] which has resulted in drug companies extensively testing potential therapeutic compounds for I_Kr_ block prior to introduction to the market.

## Role of IKr in hiPSC Cardiomyocytes

The importance of I_Kr_ in cardiac repolarization has been highlighted in several studies [11,12]. In recent years, myocytes from large animals are being phased out in favor of human induced pluripotent stem cell derived cardiac myocytes (hiPSC-CM) [13,14]. In contrast to adult myocytes, hiPSC-CMs are deficient or lack several K^+^ currents which are important for repolarization suggesting hiPSC-CMs have a reduced repolarization reserve compared to adult myocytes. These deficiencies include a negligible I_K1_ [15], a functionally absent I_to_ [16] and negligible I_Ks_ [17] suggesting these cells are immature electrophysiologically. The absence of t-tubules coupled with their small size suggests they are morphologically immature [18] indicating that results obtained in hiPSC-CM may not be translatable to the adult phenotype.

There are extensive studies detailing methods to improve the maturity level of hiPSC-CM such as plating hiPSC-CMs on a more rigid matrix [19]. Other investigators have attempted to “mature” hiPSC-CMs by expressing K^+^ current(s) that are not present at this stage of development [20]. In a previous study from our group, we enhanced I_Kr_ by pharmacological methods using several small molecule activators of K^+^ currents (such as NS3623) which have been shown to increase repolarization reserve [21,22]. The results of those studies indicated that I_Kr_ is critical during the repolarization phase of the AP and plays a major role in setting the membrane potential. Inhibition of I_Kr_ (by E-4031) resulted in a depolarization of the maximum diastolic potential (MDP) [23]. Conversely, enhancement of I_Kr_ (by NS3623) resulted in a shortening of the hiPSC-CM action potential and hyperpolarization of the MDP [22].

**Figure 1:**
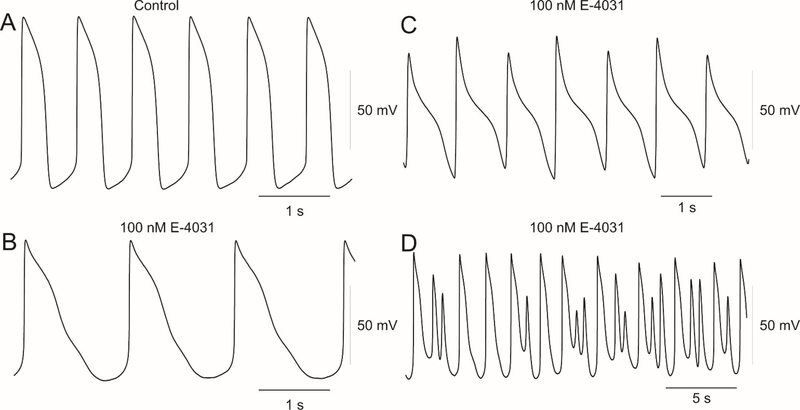
APs showing spontaneous activity (Panel A) and triangulation of the hiPSC myocyte AP following exposure to E-4031 (Panel B). Repolarization alternans (Panel C) and early afterdepolarizations (Panel D) were also observed following application of E-4031.

The role of I_Kr_ in modulating the AP duration in hiPSC and native myocytes is well established [24,25]. Tissue type is also important in determining the degree of drug-induced prolongation of the AP, presumably due to the complement of K^+^ currents present in the tissue types [4,26]. Figure 1 shows action potentials recorded from hiPSC-CMs and the changes in AP waveform and duration when exposed to low concentrations of the I_Kr_ inhibitor E-4031 (100nM). In response to 100nM E-4031, a triangulation of the AP was observed, and the duration was prolonged (Panels A-B). Prolonged exposure to E-4031 resulted in the development of repolarization alternans (Panel C) and formation of EADs (Panel D).

**Figure 2:**
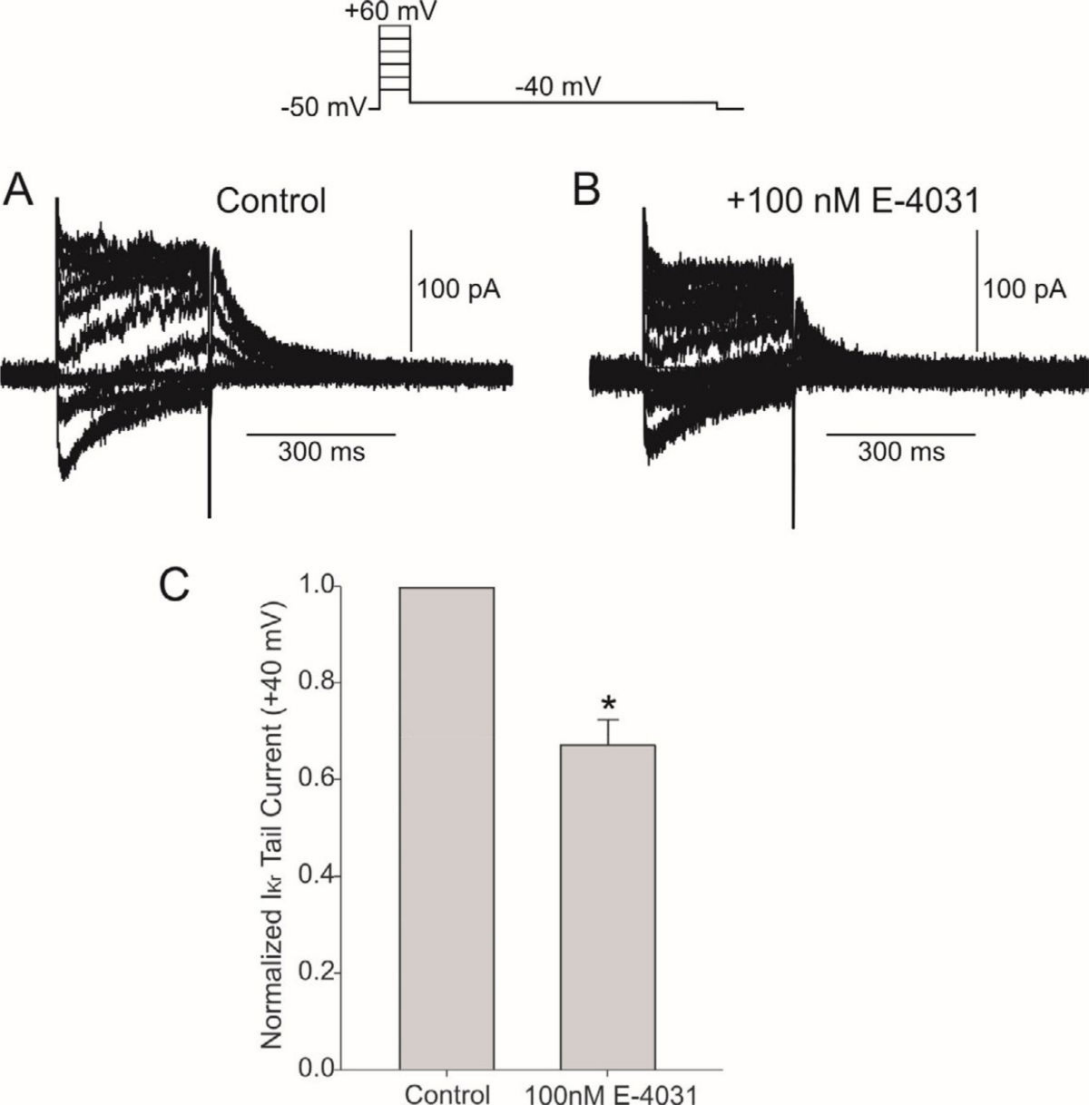
Representative I_Kr_ traces recorded from a hiPSC-CM in the absence (Panel A) and presence of the I_Kr_ inhibitor E-4031 (Panel B). The voltage clamp protocol is shown at the top of the figure. Summary data showing that 100 nM E-4031 decreased the size of I_Kr_ tail currents (Panel C). *significantly different compared to control.

We next measured the magnitude of I_Kr_ in hiPSC-CMs and the amount of I_Kr_ blockade following 100nM E-4031. Representative traces showing I_Kr_ recorded from hiPSC-CMs in the absence and presence of E-4031 (Figures 2A and 2B). Application of 100nM resulted in a 31% reduction in tail current (Figure 2C). Higher concentrations of E-4031 (5μM) resulted in no tail current demonstrating that only I_Kr_ is present under these recording conditions.

In the recent years, notable attempts were made towards developing *in silico* biophysical models of hiPSC-CMs which reasonably reproduce the experimental AP morphology and intracellular calcium dynamics of hiPSC-CMs [27,28]. Most of these models were parameterized by averaging a large amount of *in vitro* data from multiple sources in order to cover the range of variability seen in these cells. This approach addresses the limitations of earlier models which were based on inadequate data. However, the unresolved inconsistencies in experimental protocols used by the various sources have the potential to introduce unwarranted deviations in the hiPSC-CM electrophysiological parameter range as well as the generalizability of the baseline model. For example, blocking I_Kr_ by 33% resulted in 60% prolongation in APD_90_ in Koivumäki et al. model [29] whereas up to 59% I_Kr_ block produces mere 21.8 ± 10% APD_90_ prolongation in Kernik et al. [28]. Such large variations in model behavior could be attributed to the fact that the I_Kr_ formulation in Koivumäki model was based on adult ventricular myocyte kinetics [30] which was scaled by averaging experimental data from seven different sources, experimental conditions of which most were “not known”. The Kernik model I_Kr_ formulation was parameterized by averaging experimental data from four different sources. Moreover, none of these models include I_KACh_, whose channels have recently been found to be present and functional in hiPSC-CMs [31]. The inclusion of I_KACh_, allows for the investigation of the variability in the spontaneous beating frequency influenced by parasympathetic influences and/or the presence of acetylcholine which have been found to reduce the heart rate.

**Figure 3:**
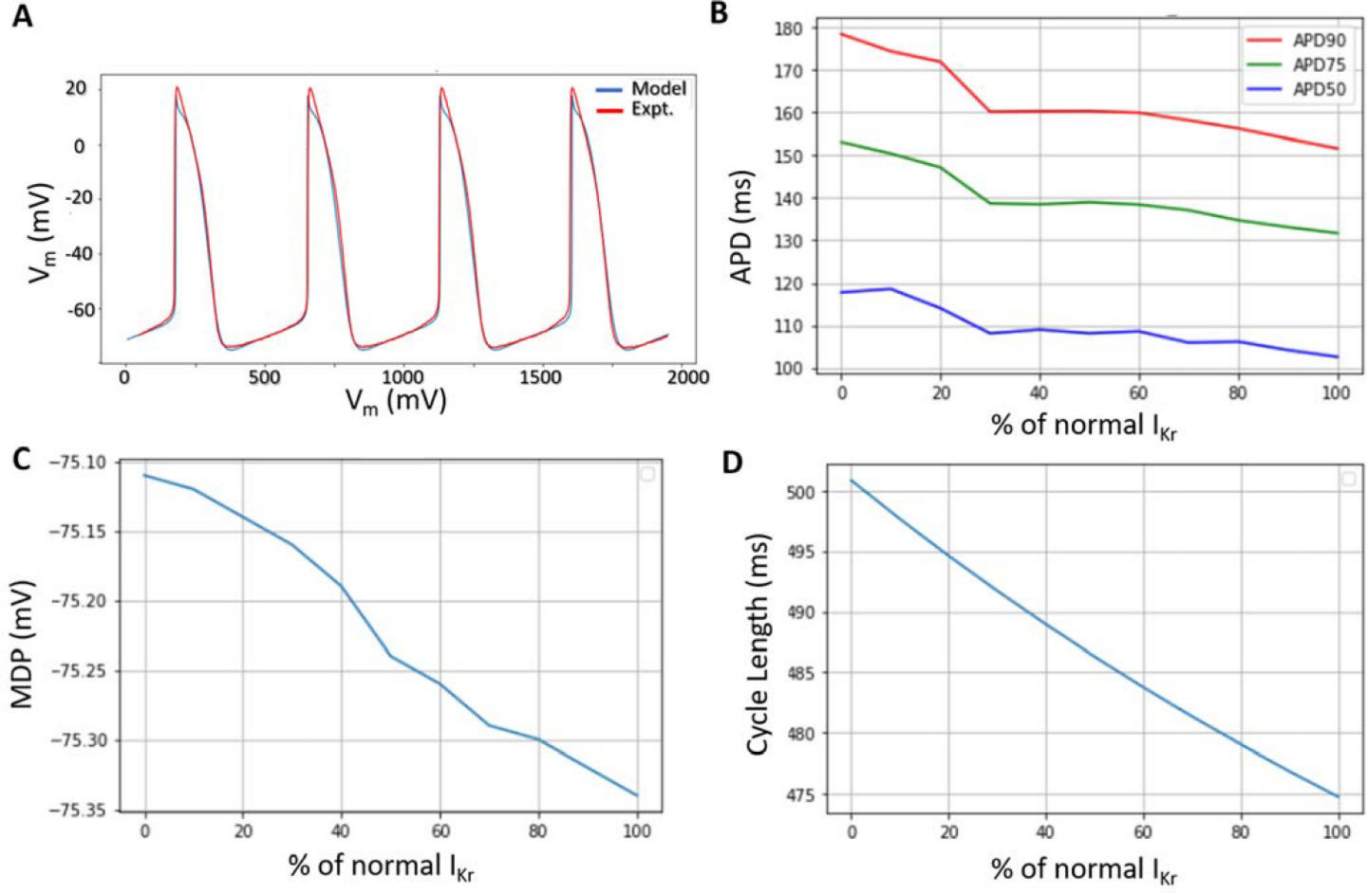
Computer model outcome. A) AP morphology and spontaneous triggering of APs in computer model (blue) compared to experimental data (red). B) AP prolongation at various levels of I_Kr_ blockade. C) Depolarization of MDP and D) increase in cycle length (CL) of spontaneous APs for varying levels of I_Kr_ block.

We implemented a genetic algorithm-based optimization method to fit the experimental I-V curves of five key currents in hiPSC-CMs to mathematical formulations from adult human atrial and sinoatrial cell models. Our aim was to utilize experimental data from a single source to maintain consistent experimental conditions and protocols as far as possible. Therefore, we utilized data acquired in our lab on the following five key currents: fast sodium current, I_Na_; transient outward potassium current, I_to_; L-type calcium current, I_CaL_; rapid delayed rectifier potassium, I_Kr_; and hyperpolarization-activated current, I_f_. The Hodgkin-Huxley style characteristic equations of current density as well as activation/inactivation gating for these currents were formulated. The parameter sets of each current formulation were combined together as chromosomes and were optimized heuristically by genetic algorithm over 100 generations [32]. The maximum conductances of the remaining ionic channels were then scaled based on recommendations from literature to reasonably reproduce the experimentally observed hiPSC-CM action potential morphology and automaticity. Our numerical model was able to accurately reproduce the experimentally recorded AP morphology of hiPSC-CMs as well as their automaticity (Figure 3A). We then studied the effects of varying I_Kr_ blockade in our model by scaling the maximum conductance of I_Kr_ (G_Kr_) from 0–100%. It was observed that the APD was prolonged by approximately 15% when I_Kr_ was blocked completely (Figure 3B). The MDP was elevated slightly as the extent of I_Kr_ block was increased (Figure 3C) and the basic cycle length of spontaneous APs was monotonically increased with the extent of I_Kr_ block (Figure 3D). Our model qualitatively reproduced the depolarization of the resting membrane potential in presence of I_Kr_ block. However, it should be noted that the model being generic, did not produce the severe effects of long-term I_Kr_ block such as triangulation of AP and alternans/EADs as observed in the experiments which warrants a more systematic *in silico* investigation.

## Summary and Conclusions

In hiPSC myocytes, I_Kr_ is important in determining the MDP and plays a role in controlling action potential duration. Inhibition of this current by low concentrations of E-4031 (100nM) results in depolarization of MDP, prolongation of the APD and development of EADs. These effects in hiPSC myocytes are in contrast to those seen in adult ventricular myocytes. In both guinea pig and rabbit ventricular myocytes, application of 10μM E-4031 resulted in about a 70ms prolongation of the action potential duration [33] and no depolarization of the membrane potential was observed. Similarly, Gintant demonstrated that 5μM E-4031 applied to canine midmyocardial cells caused a 100 ms prolongation in the APD [34]. These results highlight that micromolar concentrations of E-4031 produce modest changes in APD in adult myocytes whereas nanomolar concentration produce dramatic changes in hiPSC myocytes, namely depolarization of MDP, AP prolongation and EADs. The deficiency of several K^+^ currents important for repolarization (such as I_K1_ and I_Ks_) is likely responsible for the higher sensitivity of hiPSC-CMs to I_Kr_ inhibition. It is worth noting that canine ventricular myocytes exhibit depolarization of the membrane potential and EADs when exposed to combine I_Kr_ and I_K1_ blockade [35].

## Safety Pharmacology and the Use of hiPSC-CM to Study I_Kr_?

hiPSC-CM are utilized in many applications such as models of cardiac genetic diseases [36–38]. In addition, the utilization of hiPSC-CMs for safety pharmacology is becoming more attractive as highlighted by the proposed Comprehensive In Vitro Pro-Arrhythmia Assay (CiPA) initiative [14]. CiPA aims to examine how pharmaceutical agents bound for regulatory submission to the Food and Drug Administration will affect multiple ion channels in adult cardiac tissue as well as in stem cell derived human cardiac myocytes. This new drug testing paradigm will replace the hERG channel/Purkinje fiber assay to assess potential QT prolongation of novel compounds. The use of hiPSC-CMs would seem like the ideal experimental model in that large quantities of human cardiac cells can be generated and high throughput electrophysiological analysis can be performed. Our experimental and modeling results would suggest the hiPSC-CMs are an excellent platform for assessing cardiotoxicity as hiPSC-CMs and adult native myocytes exhibit a similar response to selective I_Kr_ blockade which may facilitate *in vitro* identification of drug-induced effects. However, since hiPSC myocytes have a low repolarization reserve compared to adult ventricular myocytes, this cell type has a greater arrhythmogenic potential due to excessive APD prolongation.
